# Supplementary material for: Differentially Expressed Genes Induced by Erythropoietin Receptor Overexpression in Rat Mammary Adenocarcinoma RAMA 37-28 Cells
Source: Int J Mol Sci. 2023 May 9;24(10):8482. doi: 10.3390/ijms24108482 (PMC10218110; doi:10.3390/ijms24108482)
Supplement: Supplementary file 1 [file ijms-24-08482-s001.zip › Supplementary Figures.pdf]

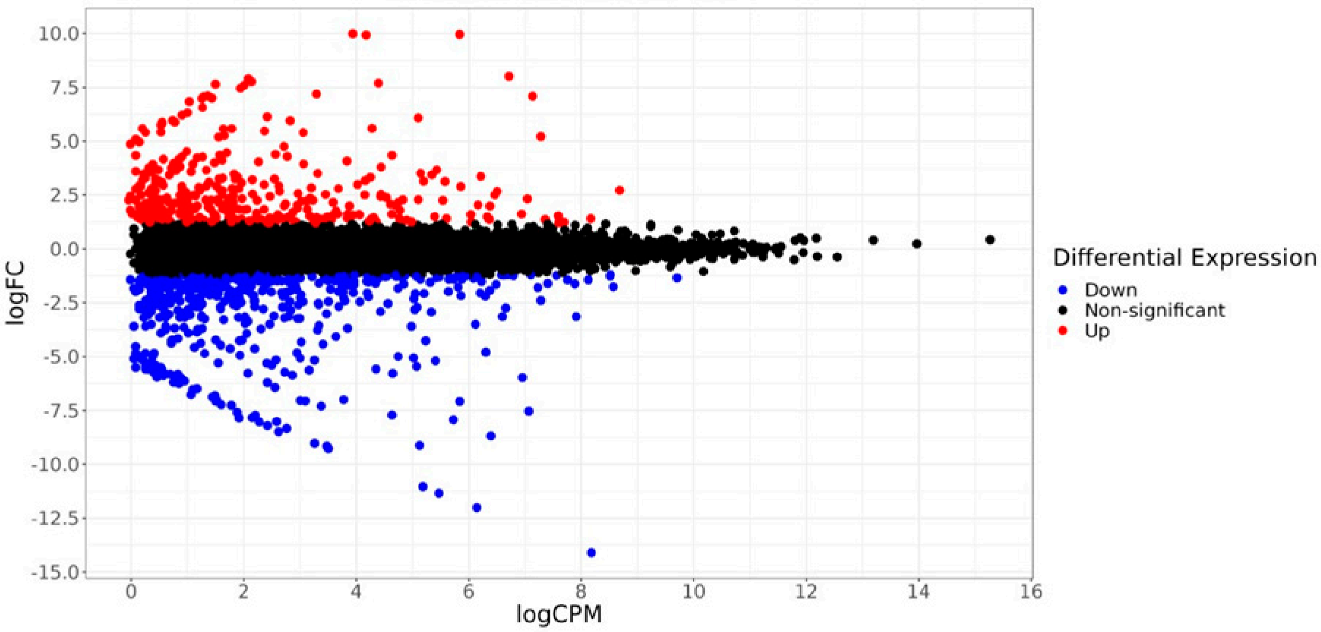

Figure S1. Venn diagram – differentialy expressed genes (DEGs) in RAMA 37-28 compared to RAMA 37 cells. Red shaded genes are upregulated and blue shaded ones are downregulated. Black color indicates no significant change in gene expression. Range of the fold change (logFC values) is presented in the scale.

**Upregulated**	**Ratio (protein expression) R3728 / R37**
**Oncomodulin (OM)** (Parvalbumin beta)	7.1
Aldehyde dehydrogenase 6 (EC 1.2.1.5)	4.7
Cornifin B (Small proline-rich protein IB) (SPR-IB)	2.6
**Gamma-synuclein** (Persyn)	2
Zyxin	2
Cation-transporting P-type ATPase B (EC 3.6.3.-)	2

**Downregulated**	**Ratio (protein expression) R3728 / R37**
**Glypican-4** precursor (K-glypican)	0.4
Prefoldin alpha subunit (GimC alpha subunit)	0.4
Citrate synthase (EC 4.1.3.7)	0.4
Zinc-carboxypeptidase precursor (EC 3.4.17.-)	0.4
Orotidine 5'-phosphate decarboxylase (EC 4.1.1.23)	0.4
Chymotrypsin BI precursor (EC 3.4.21.1)	0.4
Hypothetical protein MJ0443	0.3
Hypoxanthine-guanine phosphoribosyltransferase	0.2
Ribulose bisphosphate carboxylase large chain	0.1

Figure S2. Proteome analysis of more than 5000 peptides confirmed the altered expression of OCM2 (logFC 7.43) and SNCG (logFC 2.08) genes from the transcriptome analysis, but also revealed new DEGs and/or proteins such as ALDH6 (logFC 5.51), SPRR1B (logFC 2.64), ZYX (logFC 2.28), CTPB (logFC 4.62), HPRT (logFC −5.31) and hypothetical protein MJ0443 (logFC −3.51)

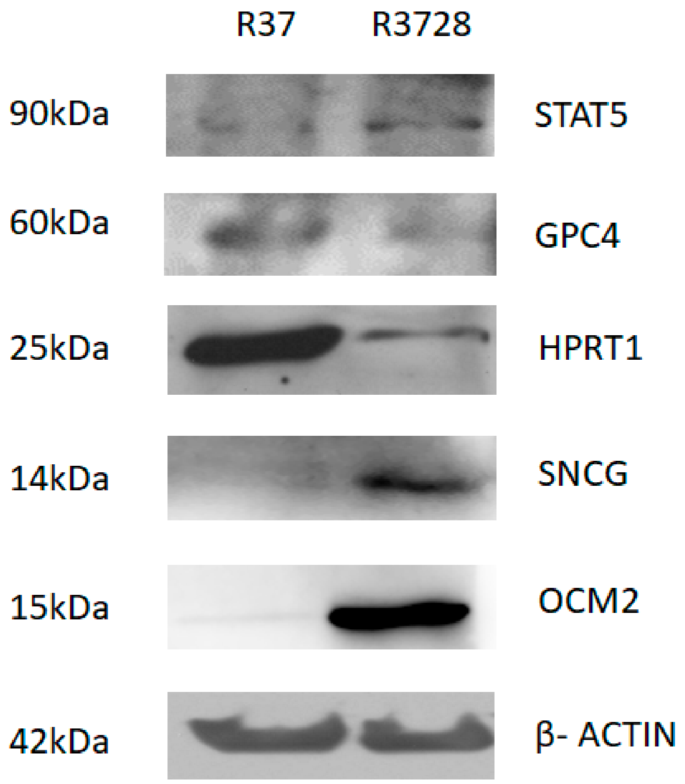

Figure S3. Western blot analysis of proteins STAT5, GPC4, HPRT1, SNCG, OCM2 and β-ACTIN in RAMA37 (R37) and RAMA 37-28 (R3728) cells.

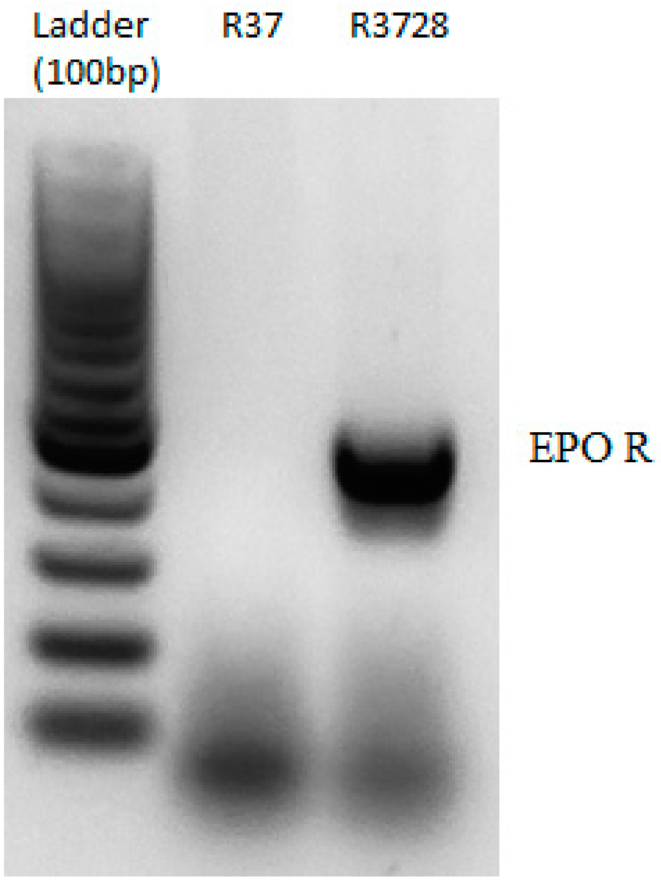

Figure S4. qRT-PCR analysis of *EPOR* in RAMA 37-28 (R3728) compared to RAMA 37 (R37) cells.

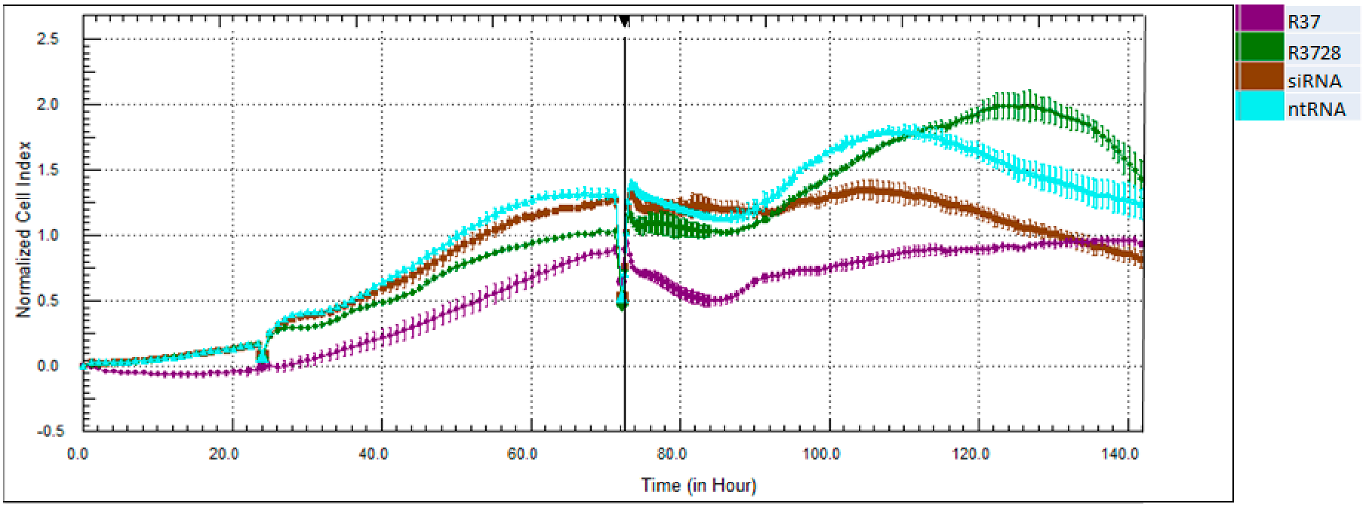

Figure S5. xCELLigence Real-Time Cell Analysis. Cell proliferation (adhesion) of RAMA 37 control cells and RAMA 37-28 cells treated with siRNA (14nM), or ntRNA (5nM) for 48 h (24 h after seeding) followed by PTX (200nM) treatment for 68 h. Cells were monitored at 60 min intervals over a 140 h monitoring period using *xCELLigence* RTCA system.
